# Health Status and Health Determinants of Older Immigrant Women in Canada: A Scoping Review

**DOI:** 10.1155/2015/393761

**Published:** 2015-07-27

**Authors:** Sepali Guruge, Birpreet Birpreet, Joan A. Samuels-Dennis

**Affiliations:** ^1^School of Nursing, Ryerson University, 350 Victoria Street, Toronto, ON, Canada M5B 2K3; ^2^School of Health Science, Humber College ITAL, 205 Humber College Boulevard, Toronto, ON, Canada M9W 5L7

## Abstract

Increasing international migration in the context of aging populations makes a comprehensive understanding of older immigrant women's health status and determinants of their health particularly urgent. Using Arksey and O'Malley's framework, we conducted a scoping review to examine the available literature on the health of older immigrant women in Canada. We searched CINAHL, PsycINFO, Embase, Medline, and Cochrane databases for the period of 1990 to 2014 for Canadian-based, peer-reviewed studies on the topic. A total of 20 articles met the inclusion criteria. These articles were divided into six areas of focus: physical health; mental health; abuse; health promotion and chronic disease prevention; barriers to healthcare access and utilization; and health beliefs, behaviours, and practices. Our results show that the health of older immigrant women is affected by the interplay of various social determinants of health including the physical and social environment; economic conditions; cultural beliefs; gendered norms; and the healthcare delivery system. Our results also revealed that older immigrant women tend to have more health problems, underutilize preventive services, such as cancer screening, and experience more difficulties in accessing healthcare services.

## 1. Background

Aging and international migration are two prevailing global trends that have changed the age and ethnic composition of populations in countries worldwide, particularly Canada, the United States, the United Kingdom, and Australia. The global share of older people (aged 60 years or over) increased from 9.2% in 1990 to 11.7% in 2013 and will continue to grow, reaching 21.1% by 2050 [1]. Between 1990 and 2013, the number of older immigrants increased from 26 to 37 million worldwide [1]. This trend that is being observed in nearly all countries around the globe is the result of several factors including declining fertility rates as well as advances in medical care and technology and public health initiatives that have reduced mortality and morbidity rates for both chronic and acute illnesses [1]. These demographics demand changes in policies and service delivery systems that address the health and illness concerns of older immigrants [2].

The number of older women exceeds that of older men in most countries worldwide. In 2013, globally, there were 85 men per 100 women in the age group 60–79 years and 61 men per 100 women in the age group 80 years or older [3]. This phenomenon is referred to as the “feminization of aging” [3]. Older men and women have different health and illness experiences: women tend to experience more illnesses, more years of disability, and more stress than men, but they tend to live longer [4]. These gender disparities have been attributed partly to genetic predisposition, physiological and hormonal differences, and socioeconomic factors [4]. Research suggests that the family roles and responsibilities (such as caregiving) of older women often reduce their ability to make decisions regarding their own health and limit their access to and use of healthcare services [5]. Clarifying what is known about the health status of older women and the factors that affect their health is important to meet the health needs of this population. Building on our current and previous work, we focused on this topic in Canada.

Older adults are the fastest-growing age group in Canada. In 2011, an estimated 5.0 million individuals in Canada were 65 years or older, and this number is expected to double in the next 25 years to 10.4 million by 2036. By 2051, approximately one in four (25%) Canadians is expected to be 65 years or older [6]. The health of Canada's older immigrant population is of great interest to clinicians, health care managers, and policymakers because older immigrants' health is an important measure of population health and is directly related to issues, such as the cost and adequacy of the Canadian healthcare system [7]. For example, Gee et al. [5] reported that recent older immigrants are usually healthier than their Canadian-born counterparts or older immigrants who have been in Canada for many years, but over time their health may decline to a level that is worse than that of Canadian-born older adults. This phenomenon, the “healthy immigrant effect,” has also been documented in Australia [8], the United Kingdom [9], and the United States [10]. It has been reported that this deterioration applied only to “non-European” immigrants [11]. Overall, this topic has begun to receive attention from health sciences researchers.

We conducted a scoping review to examine the existing literature about the health of older immigrant women in Canada. Our objectives were to (1) summarize the current knowledge about the health status of older immigrant women in Canada; (2) identify gaps in the existing knowledge; and (3) highlight implications for research, practice, and policy.

## 2. Methods

We followed the five-stage process that was proposed by Arksey and O'Malley [12] for conducting a scoping review: to identify a research question; identify studies relevant to the research question; review and select a subset of studies for inclusion in the final review; chart the information and data for the selected studies; and collate, summarize, and present the results.

Our research question for the scoping review was as follows: what is known from the existing literature about the health of older immigrant women in Canada? With the help of an experienced librarian, we searched CINAHL, Embase, Cochrane, Medline, and PsycINFO databases from January 1990 to 2014 using the following combinations of keywords: old age OR senior, elder, and older; AND immigrant, refugees, precarious, ethnic minority, visible minority, racial, and racialized; AND health AND Canada, Canadian. Inclusion criteria for articles were (1) peer-reviewed research findings; (2) being published in English or French; (3) focusing on older immigrant women's health; and (4) being based on studies conducted in Canada.

In most developed countries, including Canada, an “older adult” is defined as someone aged 65 years or older [13]. However, in many low-income countries with relatively shorter life expectancies, this definition may be expanded to include individuals aged 55 years or older [14]. For the purposes of this scoping review, we defined older adults as individuals aged 55 and older, because this is the definition generally used among our target population, which consists of a large proportion of older immigrants from low- and middle-income countries. We included studies that did not focus primarily on older women if their sample mean or median age range was 55 years or older and if at least half of the participants were women and separate results were available for this group. We defined “immigrants” as individuals who were born outside Canada (to non-Canadian parents), at some point, permanently relocated to Canada. If a study included both immigrant and Canadian-born older adults, we included it only if at least half of the study sample was born outside Canada, and separate results were available for this group.

**Figure 1:**
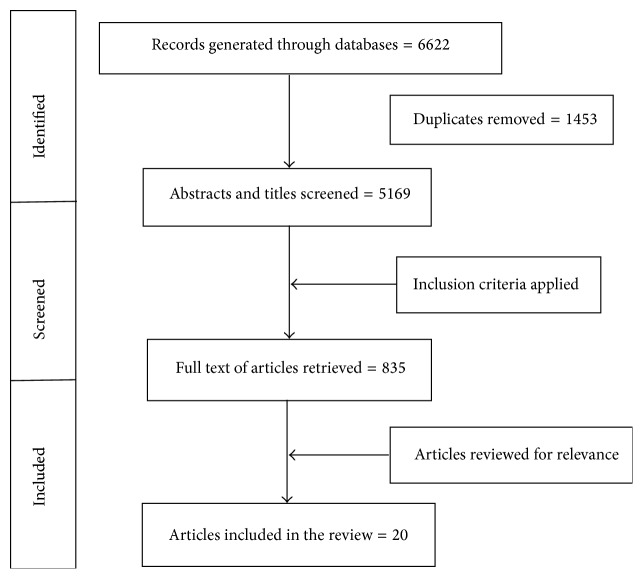
Flowchart of literature search and selection.

A total of 6622 (CINAHL: 1261; Embase: 2223; Cochrane: 312; Medline: 1923; and PsycINFO: 903) records were generated through database searches. Of these, 1453 duplicates were removed and the abstracts of remaining 5169 articles were read for the inclusion criteria. After removing 4304 articles that did not focus on older immigrants, full text of 835 articles was read to determine if they focused on the health of older immigrant women. Twenty articles met our inclusion criteria and were included in the scoping review (see Figure 1).

As per Arksey and O'Malley [12], scoping reviews are undertaken to map the extent, range, and nature of literature, so we did not assess the quality of studies included in the review. We charted the 20 articles in Microsoft Excel 2011 using the following headings: Author/s, Name of Journal, Year of Publication, and Title; Aim of the Study, Focus Area; Ethnicity or Country/Continent of Origin, Age, Sample Size, and Study Setting; Study Method, Study Design, Data Collection, and Data Analysis; and Major Findings (see Table 1).

## 3. Results

### 3.1. Characteristics of the Studies Included

Among the 20 articles selected for analysis, seven (35%) were based on data collected for one study that was conducted in seven major cities of Canada: because these articles focused on different aspects of health of older immigrant women in Canada, we included them all. The characteristics reported here, therefore, are for the 14 studies (upon which the 20 articles are based). Their sample sizes ranged from 15 to 88,447. Most studies were based in Ontario (*n* = 6; 43%), British Columbia (*n* = 4; 29%), or Alberta (*n* = 2; 15%). Two were secondary analyses based on large sets of data across multiple provinces. The place of origin of immigrant women was operationalized in different ways across the studies: “country of origin,” “country of birth,” “continent of birth,” or “ethnicity.” With the exception of three articles, all (*n* = 18; 90%) included participants from Mainland China, Hong Kong, Taiwan, and Vietnam. With the exception of one (longitudinal) study, all studies used a cross-sectional design. Most (*n* = 7; 50%) used mixed methods combining qualitative and quantitative methods, six (*n* = 6; 43%) used qualitative methods, and one (*n* = 1; 7%) used a quantitative method. We did not find any articles written in French that met our inclusion criteria.

### 3.2. Summary of the Studies

We organized the 20 articles into six focus areas: physical health (*n* = 1; 5%), mental health (*n* = 3; 15%), abuse (*n* = 1; 5%), health beliefs/behaviour/practices (*n* = 8; 40%) and health promotion and chronic disease prevention (*n* = 5; 25%), and barriers to healthcare access and utilization (*n* = 3; 15%). One paper addressed both physical and mental health.

#### 3.2.1. Physical Health

Chau and Lai [16] investigated the relationship between the size of Chinese communities and the health of older Chinese immigrants in Canada. Older Chinese immigrants residing in communities with a smaller Chinese population reported better physical health than older Chinese immigrants residing in communities with a larger Chinese population. Also, in this particular study, older men tended to score higher than older women did on physical health status scales.

#### 3.2.2. Mental Health

Lai [15] categorized the predictors of depressive symptoms among older Chinese immigrants into three types: physical health factors, circumstantial factors, and psychosocial factors. Physical health factors included general physical health; circumstantial factors included finances, length of residency in Canada, barriers to services, and living arrangement; and psychosocial factors included ethnic identity, life satisfaction, and attitudes toward aging. Lai [15] also reported that older immigrants often encounter additional service barriers, making the transition from their homeland to their life in Canada stressful. Lai [17] later reported that, compared to their male counterparts, Chinese older immigrant women are often more vulnerable, more financially disadvantaged, have fewer resources, and are less healthy physically and mentally and stressed the need for improved support services aimed at older immigrant women. Chau and Lai [16] reported that the size of a Chinese community was a significant predictor of mental health status. As is the case with physical health above, participants residing in cities with a larger Chinese population scored lower on mental health scales. Social support was not a significant predictor of mental health in larger Chinese communities.

#### 3.2.3. Abuse

Lai [18] reported that 4.5% of the study sample (*N* = 2,272) had experienced at least one incident of “maltreatment” or “neglect” within the past year: the most common forms of abuse included “being scolded, yelled at, treated impolitely, and ridiculed” (p. 338).

#### 3.2.4. Health Beliefs, Behaviours, and Practices

Lai and Surood [19] reported that the health beliefs of older Chinese adults involve beliefs about “traditional” health practices (such as “Chinese exercise”), traditional Chinese medicine, and preventive diet. MacEntee et al. [20] focused on how acculturation in Canada influenced dental beliefs and behaviours among older Chinese immigrants: they found participants used “western dentistry” in addition to traditional remedies but it was difficult for them to get relevant information about oral health care: the two main barriers to accessing western-based dental care were language and financial costs. Tieu et al. [21] compared beliefs about treatment, etiology, and prognosis about depression among older Chinese immigrants and Canadian-born participants of the same age. They found that older Chinese immigrants would benefit from health education about the symptoms of depression, its etiology, and effective treatments.

Lai et al. [22] reported that participants who identified more strongly with Chinese cultural values and beliefs reported less favourable physical health and more illnesses. Jette and Vertinsky [23] explored how older Chinese immigrant women understood and took up western biomedical beliefs about health and physical activity. They found that the health practices of participants were not influenced by the western narratives of health, rather strongly tied to cultural beliefs. Green et al. [24] assessed the use of vitamin D supplements among older adults and found that 60% of older British Columbians, including those of “Asian ethnic origin,” were using a vitamin D supplement. They also found that older women were more likely than older men to take a supplement containing vitamin D. Having a healthcare practitioner recommend a vitamin D supplement doubled the likelihood of supplement use among older immigrants, demonstrating the importance of healthcare practitioners in influencing positive health behaviours. Johnson and Garcia [25] collected dietary and physical activity profiles and explored the factors that influenced these profiles. They found that older immigrants are at a high level of nutritional risk and identified contributing factors, such as inadequate dietary consumption, tooth and mouth problems, the presence of chronic conditions that require dietary modifications, multiple medications, eating alone, and limited financial resources. Fornazzari et al. [26] reported low levels of knowledge about Alzheimer's disease among a sample of older Latin-American immigrants resulting in both normalization (as a “normal part of aging”) and stigmatization (e.g., belief that it is contagious) that may act as a barrier to timely access to health care services.

#### 3.2.5. Health Promotion and Chronic Disease Prevention

Sun et al. [27] reported lower rates of mammography screening among older Asian immigrant women in Canada as compared to Canadian-born women. They found that language differences were a key barrier to mammography screening. Todd et al. [28] explored the predictors of colon and breast cancer screening among older Chinese immigrant women and found that physician recommendation, self-efficacy, and adequate English-language proficiency affected the likelihood of cancer screening. Donnelly [29] explored the processes by which older Vietnamese immigrant women decided to engage in regular breast and cervical cancer screening: key findings were that decision-making should be shared by the patient and the physician, and outreach materials should be provided in a language that is understood by and accessible to this population. Lofters et al. [30] compared the prevalence of cervical cancer screening among older immigrant women from various countries and among Canadian-born women and found that screening rates were much lower among older women from South Asia than among Canadian-born women. Their findings suggested that income and cultural differences contributed significantly to the differences in screening rates between the two groups of women. Choudhry et al. [31] focused on health promotion among South Asian immigrant women and reported that the participants found it important to “maintain culture and tradition,” placed family needs before themselves, and survived by “being strong.”

#### 3.2.6. Barriers to Healthcare Access and Utilization

Lai and Chau [32] reported that older Chinese immigrants find accessing health services difficult due to communication barriers such as language differences, lack of cultural competence among service providers, and logistical problems in the service delivery system. Lai and Kalyniak's [33] study of predictors related to accessing annual physical examinations among older Chinese immigrants revealed that, in addition to lack of knowledge about healthcare coverage and service availability, the low rates of accessing healthcare could be related to a lack of understanding about the importance and benefits of preventive annual physical examinations. Ballantyne et al. [34] investigated medication use among a diverse group of older immigrants and found that older immigrants from China, Hong Kong, Vietnam, and Portugal often avoided discussing their self-care and alternative healthcare practices with their physicians; they also found that the participants often disagreed with the assessment and the recommended treatment and sought out and tried alternative treatments.

## 4. Discussion 

This scoping review summarizes the current literature on the health status of older immigrant women in Canada and the multiple and intersecting factors that affect this population's health. The review makes a significant contribution to the health science literature as it collates the relevant research on the topic.

Many of the studies used narrow recruitment strategies (e.g., telephone surveys) and small samples. Most also focused on one ethnic group and did not compare the results with other immigrant groups or individuals born in Canada. Other limitations of the original studies included potential loss of cultural nuances during translation and interpretation of data; lack of culturally and linguistically relevant data collection instruments; and lack of information about premigration health statuses.

In spite of these limitations, these studies revealed that older immigrant women experience health disadvantages due to intersecting factors such as social isolation, poverty, gender- and generational-specific roles, and other factors linked to the social determinants of health. The World Health Organization defines the Social Determinants of Health as the conditions in which people are born, grow, live, work, and age, including the health system [35]. The social determinants of health are a key element of Canada's framework for improving the health of its population [36].

**Figure 2:**
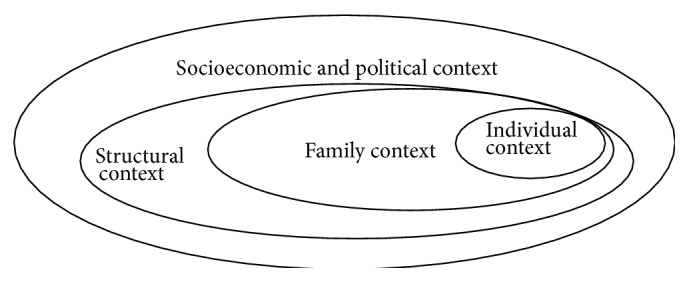
Social determinants of health.

The social determinants of health can be depicted using three circles to show how each determinant is contextually related to the others [37]. The familial and biologic context is at the centre of Figure 2, reflecting the social determinants of genetic and biological endowment, health practices and coping skills, and healthy child development. The structural context at the next level reflects socially created categories of “identity,” which affect individual and group status and, in turn, suboptimal versus optimal health. The socioeconomic and political context, depicted at the outermost level, refers to the structuring of communities and the availability of community-specific resources needed to support health. The three levels are interdependent, but the social determinants of health related to the socioeconomic and political context have the strongest influence on the health and wellbeing of individuals [37]. As is the case with all Canadians, the health priorities and experiences of older immigrant women in Canada are inherently linked to the social determinants of health. Our scoping review has identified six social determinants that strongly affect the health of older immigrant women: physical environment, social environment, economic conditions, cultural beliefs, gendered norms, and the healthcare service delivery system.

### 4.1. Socioeconomic and Political Context

Physical environment is an important determinant of health. Within the built environment, factors related to housing, the design of communities, and transportation systems can significantly influence the physical and psychological wellbeing of older immigrants [36]. Lack of or limited transportation is known to be a significant barrier for older immigrant women, in particular, in terms of access to health services [20] as well as access to supermarkets and/or “ethnic food stores” [25]. Weather has also been identified as a barrier to physical activity among older immigrant women [25].

Support from families, friends, and communities is associated with better health [36]. Lai and Kalyniak [33] identified social support as a significant enabling factor in gaining adequate access to services. Perceived social support was also associated with positive mental health outcomes in older Hispanic immigrants living in the United States [38]. Further, the size of ethnic communities also affects health and wellbeing. For example, Chau and Lai [16] found that older Chinese immigrants living in larger ethnic communities in Canadian cities reported poorer health status as compared with those living in smaller ethnic communities. They gave several possible explanations for this observation. The first involves segregation of the older Chinese immigrants from individuals of other ethnicities: the large Chinese population in major cities may mean they are unlikely to be integrated into “mainstream” Canadian society, resulting in increased “social distance” from the Canadian-born counterparts and a subsequent decline in health. Members in smaller ethnic communities tend to have stronger social bonds that lead to psychological wellbeing. Also, because smaller communities pose less “less of a threat” to the dominant group, members of smaller ethnic communities may be perceived and treated more positively. Finally, members of smaller ethnic communities may be more resilient in dealing with negative experiences, resulting in better health and functioning.

Lai and Chau [32] found that older Chinese immigrants reported difficulties in accessing services because of lack of knowledge about existing health services, communication barriers, lack of cultural competence on the part of service providers, and administrative problems in the service delivery system. Language was a significant barrier to effective communication with a physician and mammogram use among older Asian immigrant women [32]. Sun et al. [27] reported that older Asian immigrant women who spoke one of the official languages were almost three times more likely to have ever had a screening mammogram. Similarly, studies based in the United States [39] have found language as a significant barrier to access to healthcare services. Publicly insured healthcare and drug benefits reportedly increase the willingness of older immigrant women to regularly consult or attend appointments with a physician and to use “western medicines” [34]. However, more research is needed to clarify the factors affecting the health of older immigrant women, especially their access to and use of healthcare services

### 4.2. Structural Context

Our findings highlight the negative impact of socioeconomic barriers on health care utilization. Similar findings have been reported in other studies based in the United States [40, 41]. Lai [17] found that being financially stable is important to the physical and mental health of older immigrants: older immigrant women are particularly vulnerable because they have access to fewer resources. In a study of cervical cancer screening use among urban immigrants, older South Asian immigrant women with lower incomes had lower rates of screening [30]. Financial constraints have also been identified as a major barrier to healthy eating practices among older immigrants [25].

The health choices of older immigrants are influenced by their cultural beliefs and values. For example, older Chinese immigrants reportedly believed that mental health problems can be addressed without professional help, which may be one reason for their underutilization of mental health services compared with their non-Chinese counterparts [22]. Lai et al. [22] also reported that older Chinese immigrants who identify more strongly with “traditional” Chinese cultural values and beliefs have less favourable health outcomes in the postmigration context and provided two possible explanations for this finding. First, those with stronger cultural values and beliefs may find it more difficult to adapt to Canadian values and beliefs about health, health practices, and service access. Second, older immigrants often face service barriers, such as language difficulties during encounters with healthcare practitioners. Culture also appears to play a key role in neglect and abuse: Lai and Surood [19] reported that older Chinese immigrants who were more “traditional” were more likely to experience neglect or abuse. But these findings need careful examination in the context of marginalization, stigmatization, loss or devaluation of language and culture, and lack of access to culturally appropriate and safe healthcare and services [37, 42, 43].

Gender refers to the various socially determined roles, attitudes, behaviours, values, relative power, and influence that society ascribes to women and men on a differential basis [36]. Many health issues can be ascribed to gender-based social status or roles [36]. For instance, a literature review on older Asian immigrants in North America found that depression was more prevalent among older Asian women than older Asian men [43]. The intersection of gender with other social identities had a significant effect on the health outcomes. For example, being older, female, single, and poor were all associated with negative influences on health [22]. Older women's access to health services may be related to factors such as the family's financial situation and employment concerns [29]. Often, making time for themselves at the cost of their family's needs is not an option for older immigrant women [31].

### 4.3. Social Determinants of Health Not Addressed in the Literature

One area of focus absent from the existing body of health research is how employment and working conditions affect the health of older immigrant women. The recent removal of the mandatory retirement age and anecdotal evidence that older women are remaining in the workforce longer indicate the importance of assessing how work and work environments influence the health of older immigrant women. We also did not find studies that assessed literacy and education as a determinant of older immigrant women's health or those that linked child development and the childhood environment with the health statuses of older immigrant women in Canada. No studies addressed genetic predispositions and health statuses of older immigrant women in Canada.

## 5. Strengths and Limitations of This Review

Scoping reviews are, particularly, useful for topics that have not been reviewed comprehensively before [12], as is the case of health of older immigrant women in Canada. Our review focused on social determinants of health and mirrors the current political trends that guide many health programs in Canada. The factors that shape older immigrant women's health are of importance to key stakeholders around the globe. Thus, our findings can inform practice and policy changes in this area. The findings can also inform future research on the topic.

Our review has several limitations. We excluded grey literature, which can include important research conducted by community organizations. Our literature search also did not include social science databases and thus potentially miss some relevant articles. We also did not search the reference lists of the articles included in the review.

Most of the studies included in our review included immigrants from (South, East, and Southeast) Asia, in general, and Chinese immigrants, in particular. However, the Chinese are the second-largest racialized immigrant group, making up 21% of the racialized population and 4% of the total Canadian population [44], and the findings help to clarify the health issues faced by the (diverse groups of) Asian immigrants in the country. Also, Asians constitute close to 50% of immigrants to most English-speaking countries [45], thus making our findings possibly transferable beyond the Canadian context to other countries with similar rates of Asian immigrants.

## 6. Implications

### 6.1. Implications for Research

There is an urgent need for more research on various aspects of older immigrant women's health (see Table 2). Studies involving cross-cultural groups would help reveal the degree to which findings are unique to older immigrant women from a specific ethnocultural group or common across immigrant communities. Research in different regions of Canada and different countries of the world would help clarify how geographic locations and healthcare contexts in different regions may affect older immigrant women's health and access to care and services. Studies with a longitudinal design would help delineate and track health changes, the aging process, and sociocultural changes among older immigrant women and their access to services over time. Other methodological issues to consider in future research include more emphasis on comparative research, larger sample sizes, inclusion of older immigrants from different immigration categories (such as refugee, precarious status and family sponsored), and more comprehensive recruitment strategies. An intersectionality approach [46] to examine the interactions of migration, gender, race, ethnicity, and other dimensions of social identity with health could also help further address the knowledge gap.

### 6.2. Implications for Practice

Overall, our findings suggest that the Canadian healthcare system fails to respond to the health and illness concerns of older immigrant women (see Table 2). First, it lacks holistic and supportive programs that could help older immigrant women cope with postmigration and (re)settlement challenges and achieve a sense of health and wellbeing. Second, services must incorporate community values and strengths, recognize social inequities, and implement new models of collaborative and integrated care. The healthcare and social service sectors need to work together to address the health needs of older immigrant women. There is a need for innovative community outreach strategies that engage key community leaders, to help clarify the barriers in accessing healthcare services. Healthcare practitioners need ongoing training to develop their cross-cultural communication and counselling skills. It is also important to strengthen the curricula of health professions to ensure that graduates in practical disciplines such as nursing are well equipped with the cross-cultural communication skills required for working with culturally diverse older immigrants. Also, with an increasingly aging population, demand for workers to provide care for the elderly population will increase. However, increasing the supply of healthcare providers alone is not a solution; there has to be a key focus on preventive healthcare.

### 6.3. Implications for Policy

Policy and systematic changes are needed to overcome many health and social challenges faced by older immigrant women (see Table 3). When policy recommendations were made, most articles referred to income security as a way to ensure the health of older immigrant women. A number of articles recommended policy changes that would allow for adequate access to a monthly income, rental/mortgage supplements, and extended health benefits, such as dental care. Some referred to the need for resource distribution policies to target the development of formal social support networks, because being surrounded by a large immigrant community does not always translate to social connectedness. One recommendation that was common to several articles was to promote social inclusion of older immigrant women to encourage their physical and social involvement in community life. Addressing abuse of older immigrant women should also be a key policy focus which must address the larger socioeconomic and cultural contexts that contribute to the vulnerability of older immigrant women [47]. Public policies, programs, and services for older immigrant women should extend beyond the health sector and involve collaboration with other sectors, and approaches should be multipronged to incorporate education, awareness-raising, and community-based initiatives.

## 7. Conclusion

The socioeconomic and political context in which older immigrant women live, work, and grow old has significant implications for their health and wellbeing. This scoping review suggests that older immigrant women, when compared with Canadian-born women, are more vulnerable to poor health, more financially disadvantaged, and face a greater number of barriers when accessing the services needed to maintain and/or promote their health. The findings of this review suggest that Canada's health service system is ill equipped to address the health and illness concerns of this population. Specifically, our findings suggest the need to develop healthcare service delivery approaches that account for the cultural, generational, and gender-based norms of this population. Additionally, improving older immigrant women's health will require innovative community outreach strategies that engage older immigrant women, key community leaders, family members, and other stakeholders across sectors to address barriers to social inclusion, income security, stable housing, healthy relationships, and resources to access health care which will lead to individual transformation, community development, and policy change.

## Figures and Tables

**Table 1:** Summary of charted data.

Author information	Sample information	Research design	Focus area
Lai (2004) [15]	*Ethnicity or country/continent of birth/origin*: Mainland China *Age*: 65+ years *Sample size*: 444 *Location*: Victoria, Vancouver, Calgary, Edmonton, Winnipeg, Toronto, and Montreal	*Method*: mixed *Design*: cross-sectional *Collection*: face-to-face interview *Analysis*: bivariate association using Mann-Whitney test, Kruskal-Wallis test, and Spearman's correlation test	Mental health

Chau and Lai (2011) [16]	*Ethnicity or country/continent of birth/origin*: Mainland China *Age*: 55+ years *Sample size*: 2,272 *Location*: Victoria, Vancouver, Calgary, Edmonton, Winnipeg, Toronto, and Montreal	*Method*: mixed *Design*: cross-sectional *Collection*: face-to-face interview *Analysis*: hierarchical multiple regression analysis	Physical and mental health

Lai (2011a) [17]	*Ethnicity or country/continent of birth/origin*: China *Age*: 65+ years *Sample size*: 151 *Location*: Calgary	*Method*: mixed *Design*: cross-sectional *Collection*: telephone survey *Analysis*: multiple regression analysis	Mental health

Lai (2011b) [18]	*Ethnicity or country/continent of birth/origin*: Mainland China, Hong Kong, Taiwan, Vietnam, and other Southeast Asian countries *Age*: 55+ years *Sample size*: 2,272 *Location*: Victoria, Vancouver, Calgary, Edmonton, Winnipeg, Toronto, and Montreal	*Method*: mixed *Design*: cross-sectional *Collection*: face-to-face interview *Analysis*: hierarchical logistic regression analysis	Abuse

Lai and Surood (2009) [19]	*Ethnicity or country/continent of birth/origin*: Mainland China, Hong Kong, Taiwan, Vietnam, and other Southeast Asian countries *Age*: 55+ years *Sample size*: 2,272 *Location*: Victoria, Vancouver, Calgary, Edmonton, Winnipeg, Toronto, and Montreal	*Method*: mixed *Design*: cross-sectional *Collection*: face-to-face interview *Analysis*: multiple stepwise regression analysis	Health beliefs

MacEntee et al. (2012) [20]	*Ethnicity or country/continent of birth/origin*: China, Hong Kong *Age*: 65+ years *Sample size*: 51 *Location*: Vancouver and Melbourne	*Method*: qualitative *Design*: cross-sectional *Collection*: focus groups *Analysis*: thematic analysis	Health beliefs

Tieu et al. (2010) [21]	*Ethnicity or country/continent of birth/origin*: China *Age*: 55–87 years *Sample size*: 53 *Location*: Calgary	*Method*: mixed *Design*: cross-sectional *Collection*: interview *Analysis*: multiple chi-square analyses	Health beliefs

Lai et al. (2007) [22]	*Ethnicity or country/continent of birth/origin*: Mainland China, Hong Kong, Taiwan, Vietnam, and other Southeast Asian countries. *Age*: 55+ years *Sample size*: 2,272 *Location*: Victoria, Vancouver, Calgary, Edmonton, Winnipeg, Toronto, and Montreal	*Method*: mixed *Design*: cross-sectional *Collection*: face-to-face interview *Analysis*: hierarchical multiple regression using stepwise method	Health beliefs

Jette and Vertinsky (2011) [23]	*Ethnicity or country/continent of birth/origin*: China *Age*: 65+ *Sample size*: 15 *Location*: Vancouver	*Method*: qualitative *Design*: cross-sectional *Collection*: in-depth semistructured interviews *Analysis*: thematic analysis	Health behaviour

Green et al. (2010) [24]	*Ethnicity or country/continent of birth/origin*: European and Asian *Age*: 50+ years *Sample size*: 1,996 *Location*: British Columbia	*Method*: mixed *Design*: cross-sectional *Collection*: phone-administered survey *Analysis*: multivariate logistic regression	Health behaviour

Johnson and Garcia (2003) [25]	*Ethnicity or country/continent of birth/origin*: Cambodian, Latin-American, Vietnamese, and Polish *Age*: mean age = 68 ± 6 years *Sample size*: 54 *Location*: London, Ontario	*Method*: qualitative *Design*: cross-sectional *Collection*: questionnaire and questionnaire-guided interviews *Analysis*: descriptive analysis using SPSS	Health behaviour

Fornazzari et al. (2009) [26]	*Ethnicity or country/continent of birth/origin*: Latin America *Age*: 55+ years *Sample size*: 125 *Location*: Greater Toronto Area	*Method*: mixed *Design*: cross-sectional *Collection*: questionnaires and scales *Analysis*: descriptive analysis using SPSS	Health beliefs

Sun et al. (2010) [27]	*Ethnicity or country/continent of birth/origin*: Asia *Age*: 50–69 years *Sample size*: 508 *Location*: Canada	*Method*: mixed *Design*: cross-sectional *Collection*: data from the Canadian Community Health Survey cycle 2.1 (2003) *Analysis*: multivariate logistic regression analyses	Health promotion and chronic disease prevention

Todd et al. (2011) [28]	*Ethnicity or country/continent of birth/origin*: China *Age*: 50+ years (mean 63.61) *Sample size*: 103 *Location*: Ontario	*Method*: mixed *Design*: cross-sectional *Collection*: interviews *Analysis*: Fisher's exact tests and independent sample *t* tests, multivariate logistic regression	Health promotion and chronic disease prevention

Donnelly (2006) [29]	*Ethnicity or country/continent of birth/origin*: Vietnam *Age*: 49–78 years *Sample size*: 15 *Location*: Western Canadian city	*Method*: qualitative *Design*: cross-sectional *Collection*: interviews; semistructured questionnaire using open-ended questions *Analysis*: qualitative data/thematic analysis	Health promotion and chronic disease prevention

Lofters et al. (2010) [30]	*Ethnicity or country/continent of birth/origin*: East Asia and Pacific, Eastern Europe and Central Asia, Latin America and Caribbean, Middle East and North Africa, South Asia, Sub-Saharan Africa, USA, Australia and New Zealand, and Western Europe *Age*: 50–66 years (mean 56.2) *Sample size*: 88,447 *Location*: Ontario	*Method*: quantitative *Design*: population-based cohort study *Collection*: Landed Immigrant Data System *Analysis*: multivariate Poisson regression	Health promotion and chronic disease prevention

Choudhry et al. (2002) [31]	*Ethnicity or country/continent of birth/origin*: South Asian *Age*: 58 and 68 years and 40 and 60 years *Sample size*: 13 *Location*: in and around Toronto	*Method*: qualitative *Design*: cross-sectional *Collection*: focus group discussions *Analysis*: reflexive and dialectical critique	Health promotion and chronic disease prevention

Lai and Chau (2007) [32]	*Ethnicity or country/continent of birth/origin*: Mainland China, Hong Kong, Taiwan, Vietnam, and other Southeast Asian countries. *Age*: 55+ years *Sample size*: 2,272 *Location*: Victoria, Vancouver, Calgary, Edmonton, Winnipeg, Toronto, and Montreal	*Method*: mixed *Design*: cross-sectional *Collection*: face-to-face interview *Analysis*: multiple regression analysis	Healthcare access

Lai and Kalyniak (2005) [33]	*Ethnicity or country/continent of birth/origin*: Mainland China, Hong Kong, Taiwan, Vietnam, and other Southeast Asian countries. *Age*: 55+ years *Sample size*: 2,272 *Location*: Victoria, Vancouver, Calgary, Edmonton, Winnipeg, Toronto, and Montreal	*Method*: mixed *Design*: cross-sectional *Collection*: face-to-face interview *Analysis*: hierarchical logistic regression analysis	Healthcare utilization

Ballantyne et al. (2011) [34]	*Ethnicity or country/continent of birth/origin*: China, Hong Kong, Vietnam, and Portugal *Age*: 65+ years *Sample size*: 30 *Location*: Toronto	*Method*: qualitative *Design*: cross-sectional *Collection*: interview *Analysis*: inductive content analysis	Healthcare service utilization

**Table 2:** Key implications from selected studies.

List of studies	Key implications
Lai (2004) [15]	Adequate community support services and networks
Chau and Lai (2011) [16]	Further research to examine the residual confounding effects of the socioeconomic and political environment related to racism and various forms of discrimination in large urban centres
Lai (2011a) [17]	Further research on the influence of cross-cultural differences on health
Lai (2011b) [18]	Community education
Lai and Surood (2009) [19]	Longitudinal studies to clarify the effects of aging and acculturation on health values and beliefs
Tieu et al. (2010) [21]	Mental health education integrated into supports and services
Lai et al. (2007) [22]	Service delivery with attention to individuals' cultural health practices, using an approach that is respectful and nonjudgmental
Johnson and Garcia (2003) [23]	More research about adequate dietary intake and regular physical activity of older immigrants
Fornazzari et al. (2009) [26]	Longitudinal studies about health promotion initiatives
Sun et al. (2010) [27]	Culturally and linguistically appropriate education programs about breast cancer risk and mammography screening and the role of the physician in influencing older immigrant women's mammography screening behaviours
Donnelly (2006) [29]	Research about the influence of culture, social, political, historical, and economic background on cancer screening
Lofters et al. (2010) [30]	Research about cultural barriers to cancer screening
Lai and Chau (2007) [32]	Local health agencies and community health practitioners working more closely to design culturally appropriate methods for health information and promotion activities
Lai and Kalyniak (2005) [33]	Family physicians' proactive engagement in health promotion and local health regions and with community health practitioners

**Table 3:** Overview of the policy recommendations offered by selected studies.

List of studies	Key implications
Lai (2004) [15]	*Policy focus*: income security among Chinese immigrants (i) 17.3% of the older Chinese from Mainland China had an inadequate monthly income (<$500/mth) that, in turn, negatively affected all aspects of their health. (ii) Current sponsorship regulation restricts access to government pension benefits within the first ten years after their arrival in Canada. *Recommendation*: policies have to be instituted to help older immigrants access and/or maintain an adequate level of financial stability to protect society from the probable social and fiscal cost of physical and mental health problems that are a direct result of poverty.

Chau and Lai (2011) [16]	*Policy focus*: social support and networks (i) The presence of a larger ethnic community size does not necessarily enhance individual health and wellbeing. (ii) Older immigrants from ethnically diverse populations cannot rely solely on their immediate and community social networks for support to meet their health and wellbeing needs. *Recommendation*: the development of formal social support networks that can attend in culturally safe ways to the needs of ethnically diverse groups is essential to the health and wellbeing of older immigrant women.

Lai (2011a) [17]	*Policy focus*: income security among older Chinese immigrants *Recommendation*: there is a need for policies that will extend income security benefits to realistically account for the nutritional and rent/mortgage supplement needs of older immigrant women.

Lai (2011b) [18]	*Policy focus*: abuse of older immigrants Length of residency in Canada is a significant correlate of neglect and abuse, with long-term immigrants more likely to experience greater levels of abuse and neglect. *Recommendation*: practice policies should evolve to extend screening for abuse and neglect among older immigrant women. There is a need for policies that will support the development of shelters for older immigrant women who may be exposed to various forms of family and even community violence.

MacEntee et al. (2012) [20]	*Policy focus*: income security and dental health *Recommendation*: there is a need for policies that will increase access to routine and emergency dental care particularly among older immigrant women.

Lai et al. (2007) [22]	*Policy focus*: income security *Recommendation*: there is a need for policies that strategically focus on enhancing the health of older immigrant women who are unmarried and who are living in poverty.

Sun et al. (2010) [27]	*Policy focus*: health promotion and illness prevention *Recommendation*: practice and resource distribution policies that target breast cancer prevention need to address culturally safe ways to recruit and treat Asian immigrant women.

Lofters et al. (2010) [30]	*Policy focus*: health promotion and illness prevention *Recommendation*: practice and resource distribution policies that target cervical cancer screening need to address culturally safe ways to recruit and treat older immigrant women living in the lowest-income neighborhoods where service may be absent or where acculturation is likely to affect service uptake.

Lai and Chau (2007) [32]	*Policy focus*: health service access and effectiveness *Recommendation*: enduring and productive changes to improve racialized communities' experience with the healthcare system require the development of equitable and culturally competent systems that respect the rights of culturally diverse populations. Policies that attend to structural changes are needed to ensure that older immigrants are served in a way that prioritizes cultural safety social equity versus equality.
